# Fabrication and modulation of flexible electromagnetic metamaterials

**DOI:** 10.1038/s41378-024-00806-1

**Published:** 2025-01-20

**Authors:** Yanshuo Feng, Misheng Liang, Xiaoguang Zhao, Rui You

**Affiliations:** 1 School of Instrument Science and Opto-Electronics Engineering, Beijing Information Science and Technology University, 100192 Beijing, China; 2 Laboratory of Intelligent Microsystems, Beijing Information Science and Technology University, 100192 Beijing, China; 3 Department of Precision Instrument, Tsinghua University, 100084 Beijing, China; 4 Beijing Future Chip Technology Advanced Innovation Center, 100192 Beijing, China

**Keywords:** Nanoparticles, Nanophotonics and plasmonics

## Abstract

Flexible electromagnetic metamaterials are a potential candidate for the ideal material for electromagnetic control due to their unique physical properties and structure. Flexible electromagnetic metamaterials can be designed to exhibit specific responses to electromagnetic waves within a particular frequency range. Research shows that flexible electromagnetic metamaterials exhibit significant electromagnetic control characteristics in microwave, terahertz, infrared and other frequency bands. It has a wide range of applications in the fields of electromagnetic wave absorption and stealth, antennas and microwave devices, communication information and other fields. In this review, the currently popular fabrication methods of flexible electromagnetic metamaterials are first summarized, highlighting the electromagnetic modulation capability in different frequency bands. Then, the applications of flexible electromagnetic metamaterials in four aspects, namely electromagnetic stealth, temperature modulation, electromagnetic shielding, and wearable sensors, are elaborated and summarized in detail. In addition, this review also discusses the shortcomings and limitations of flexible electromagnetic metamaterials for electromagnetic control. Finally, the conclusion and perspective of the electromagnetic properties of flexible electromagnetic metamaterials are presented.

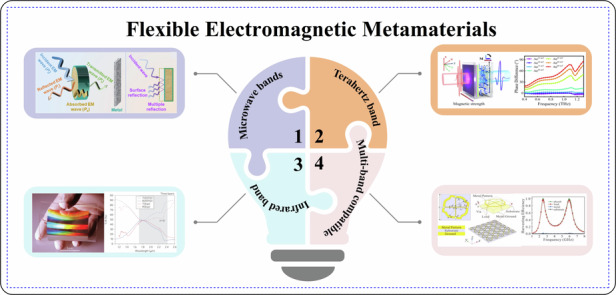

## Introduction

Since the beginning of the 21st century, electromagnetic metamaterials have received special attention from researchers as novel research products. In 2008, Landy et al. proposed the term ‘electromagnetic metamaterials’^1^, as a synthetic material, electromagnetic metamaterials have different physical properties than natural materials. It usually refers to a new type of artificial composite material formed by artificial ‘atoms’ arranged periodically or non-periodically on the sub-wavelength scale, with excellent specific properties and functions. Electromagnetic metamaterials possess specific electromagnetic properties that natural materials lack, such as negative magnetic permeability, dielectric constant, and refractive index^2,3^. These characteristics can achieve specific electromagnetic behaviors in different frequency ranges through the design and adjustment of its internal structure, and significantly enhance the means and methods of controlling electromagnetic waves. Electromagnetic metamaterials can overcome the limitations of the narrow absorption bandwidth, low absorption intensity^4^, and single absorption range that exist in current conventional metamaterials and has the characteristics of ultrahigh absorption and phase control. With the continuous development of technology, flexible electromagnetic metamaterials have shown great potential in the field of electromagnetism. Flexible electromagnetic metamaterials are composed of flexible substrates and materials that can be bent, folded, and stretched in arbitrary ways, and can adapt to a variety of complex curved surface shapes. Furthermore, flexible electromagnetic metamaterials exhibit excellent mechanical flexibility and durability, enabling them to return to their original shape after being subjected to mechanical stress. And its electromagnetic properties can be dynamically adjusted through mechanical deformation, allowing greater design flexibility. For instance, the resonance frequency and electromagnetic response characteristics of flexible metamaterials can be altered by bending or stretching them, demonstrating excellent electromagnetic control characteristics in microwave, terahertz, infrared, and multi-band frequency ranges. Flexible electromagnetic metamaterials can be controlled over a wide frequency range. It retains the excellent electromagnetic properties of traditional metamaterials. They are widely used in electromagnetic wave absorption^5,6^, stealth^7^, and antennas^8^, as well as in microwave devices^9,10^, communications^11,12^, information technology^13^, solar energy^14^, and photovoltaic technology^15,16^ and other fields.

With the continuous advancement of science and technology, flexible electromagnetic metamaterials have demonstrated increasingly impressive properties. However, the micro-nano fabrication technology of these materials remains a key limiting factor in their development. Therefore, it remains essential to identify a straightforward, convenient, flexible, and controllable processing method to streamline the structural size of flexible electromagnetic metamaterials^17–19^. By utilizing simple micro-nano processing techniques, the size and structure of these materials can be optimized, enabling electromagnetic control in various wavelength bands such as microwave, terahertz, and infrared. This optimization can enhance the absorption performance of flexible electromagnetic metamaterials. And it has great potential for future scientific and technological progress and practical engineering applications. Currently, existing articles only summarize flexible electromagnetic metamaterials from a single perspective. This article summarizes the preparation, electromagnetic regulation and current extensive application systems of flexible electromagnetic metamaterials. It provides a more comprehensive perspective for understanding flexible electromagnetic metamaterials.

**Fig. 1:**
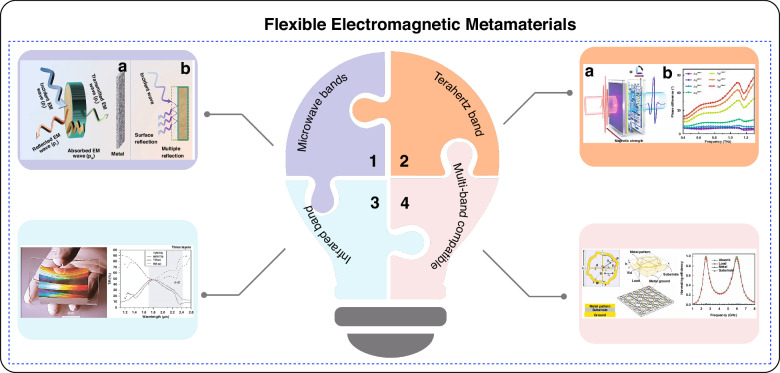
Modulation capabilities of flexible electromagnetic metamaterials in different wavelength bands and their applications

Herein, this review first introduces the current development status of micro-nano processing technology of flexible electromagnetic metamaterials. It summarizes and demonstrates the common processing methods of current flexible electromagnetic metamaterials. And conducts an in-depth discussion on its mechanism and principle. It then explores the electromagnetic modulation properties of these materials across different frequency ranges, such as microwave, terahertz, infrared, and multi-band compatibility. Figure 1 illustrates the modulation effects of flexible electromagnetic metamaterials in various wavelength bands. Furthermore, the article introduces the current applications of these materials in the fields of electromagnetic shielding, temperature regulation, anti-reflection, wearable sensors, etc., and summarizes the main challenges and prospects for future development.

## Flexible electromagnetic metamaterial manufacturing technology

Micro-nano processing technologies can be roughly divided into ‘Top-down’^20^ or ‘Bottom-up’^21^. ‘Top-down’ micro and nanofabrication are traditional fabrication methods that gradually reduce the size from the macroscopic scale to achieve the desired micro- and nanostructures^22,23^. A counterpart to this is bottom-up micro-nano-fabrication, a strategy based on the self-organization or self-assembly of materials^24^. The desired structure is assembled from basic building blocks, such as molecules and nanoparticles, by chemical synthesis, biosynthesis, or other self-assembly techniques. The scalability and efficiency of this approach make it suitable for nanomaterial preparation and nano-assembly. Micro/nanofabrication methods complement each other, each with advantages and disadvantages. Overall, micro/nanofabrication technology has significant advantages in the fabrication of flexible electromagnetic metamaterials and has a wide range of applications^10,25^.

**Fig. 2:**
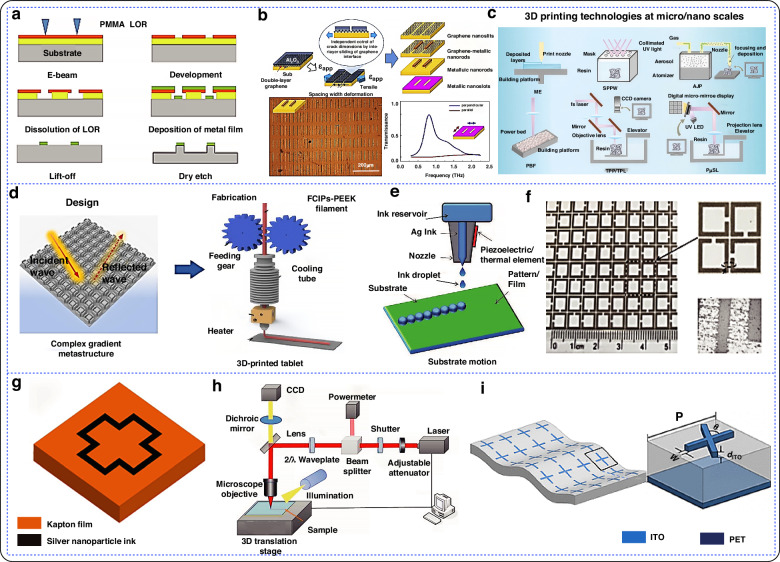
Top-down micro-nanofabrication fabrication methods. **a** Schematic diagram of the process flow of nanoimprint processing^34^. Copyright 2015 Springer Nature. **b** Fabrication of nano-antennas using photolithography^38^. Copyright 2020 Elsevier. **c** 3D printing technologies at micro/nano-scales^42^. Copyright 2023 John Wiley and Sons. **d** Preparation of complex gradient composite transfer structures using a three-dimensional printer^44^. Copyright 2021 Elsevier. **e** Schematic illustration of inkjet printing technology^29^. Copyright 2022 MDPI. **f** Geometric shapes produced by inkjet printing^48^. Copyright 2019 Springer Nature. **g** Fabrication of flexible metamaterial films using a nanoimprinting process^49^. Copyright 2016 Springer Nature. **h** Schematic diagram of laser direct writing processing system^50^. **i** Preparation of flexible transparent terahertz MMA using femtosecond laser direct writing process^56^. Copyright 2020 Optics Express

‘Top-down’ fabrication is a technical process for the fabrication of flexible electromagnetic metamaterials at the microscale. The related processes involve the gradual reduction and construction of the metamaterials structure from macroscopic objects until the required microscale is achieved. At the core of the ‘top-down’ method is the gradual reduction of the macro scale to the required micro scale. Common technologies used in this field include nanoimprint lithography (NIL)^26^, 3D printing^27^, inkjet printing technology^28,29^ and femtosecond laser direct writing (FLDW) technology^30^. Figure 2 shows a common top-down process for processing flexible electromagnetic metamaterials.

NIL^26,31^ is a technique used to create small structures on the surface of flexible electromagnetic metamaterials^32^. Chou et al. first proposed this method in 1995^33^. The NIL process is straightforward. First, a polymer film is applied to the substrate using spin coating or dripping/inkjet techniques. Then, a template with the desired pattern is pressed onto the polymer film under specific pressure. The polymer is then cured using either heat or ultraviolet light. Finally, the template is removed, leaving the corresponding micro-nano structure on the flexible polymer^34^. These structures, much smaller than the wavelength of electromagnetic waves^35^, modify the propagation characteristics and behavior of the waves^36,37^. In 2012, Jong et al. fabricated plasma-metal-insulator-metal large-area flexible metamaterials thin films by nanoimprinting^38^. Subwavelength structures can be continuously patterned on flexible substrates such as metal wafer arrays using this technique. In 2019, Won et al. proposed a method for preparing graphene-cracked metamaterial using lithography^39^.

3D printing, also known as additive manufacturing, is one of the most promising cutting-edge technologies in the world^40,41^. It has emerged as an innovative method for fabricating flexible electromagnetic metamaterials, enabling control over material structures at both the micro and macro levels. This technology simplifies the manufacturing process by constructing structures layer by layer, avoiding cumbersome traditional methods. It also offers greater flexibility in creating arbitrary topological structures. The basic principles of 3D printing involve modeling, slicing, and printing^42,43^. The slicing software then cuts the model into multiple layers and generates a printing path. Finally, the 3D printer builds the material layer by layer according to the specified path, completing the print. In 2021, Duan et al. used 3D printing technology to create a complex gradient metamaterial. By optimizing its structural parameters, its bandwidth absorption and reflectivity intensity were enhanced ^44^.

Inkjet printing is a low-cost, efficient, and high-precision digital printing process that does not require masks or templates. By editing bitmaps, ink can be printed into complex patterns. This method is highly compatible with various substrates, making it widely used in the fabrication of flexible electronic and wearable devices^28,29^. Depending on the processing needs, the inkjet printing process has evolved into two modes: continuous inkjet printing and line scanning inkjet printing. In the continuous inkjet printing mode, a continuous and unidirectional nozzle trajectory is used to create stretchable conductors with pre-designed structures, such as spiral or serpentine patterns. The line scanning mode, on the other hand, employs linear scanning with a reciprocating nozzle to perform more complex micromachining on flexible surfaces with small diameters and large curvatures. In both modes, ink is ejected onto the substrate to form the desired pattern or structure^45^. This technique allows for the precise creation of electromagnetic material structures by controlling the movement of the inkjet head and the flow of ink^46,47^. In 2019, Assimon et al. presented a novel electromagnetic metamaterials absorber that is flexible, polarization insensitive, wide-angle, and ultra-broadband^48^. In 2016, Lee et al. designed a flexible metamaterial absorber (MMA) using inkjet printing techniques^49^. At a frequency of 0.1 THz, the MM absorber achieved an absorption rate of 93.5%.

FLDW enables precise control at the micrometer and nanometer scales^30,50^. This technique is a powerful 3D “cold processing” method characterized by an ultra-short pulse duration (10^-15^ m/s) and extremely high peak power (10^14^ W/cm^2^). These properties enable cross-scale micro-nano processing across various materials^51,52^. The interaction between femtosecond laser and materials exhibits strong non-linear and non-equilibrium characteristics. When the laser interacts with matter, electrons are rapidly heated, but the energy is not immediately transferred to the lattice. As a result, the thermal effect on the material is minimal^53–55^. Additionally, due to the influence of nonlinear processes, the modification area is confined to the laser’s focal point and its immediate surroundings. This allows the laser to pass through the surface of transparent materials, enabling true three-dimensional processing. In 2020, Xiao et al. fabricated flexible electromagnetic metamaterials with different arm lengths by direct femtosecond laser etching and upper indium tin oxide (ITO) cross-shaped metamaterials with different arm lengths^56^.

**Fig. 3:**
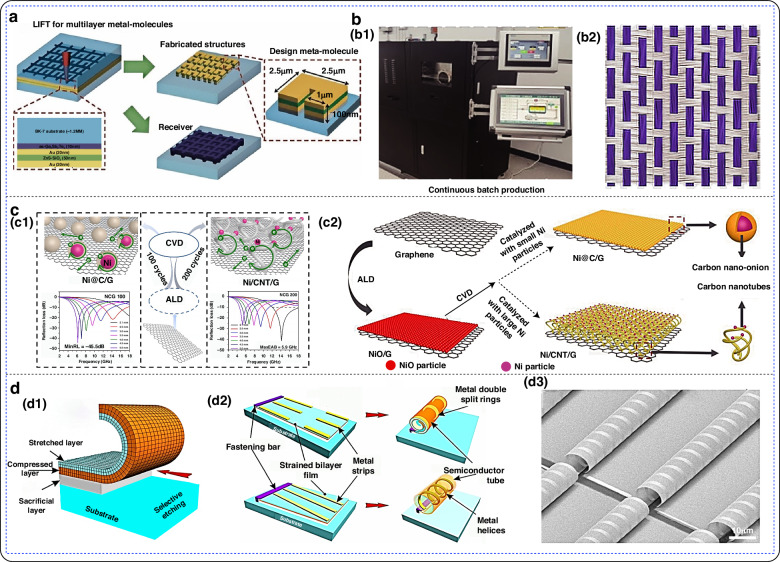
Bottom-up approach to micro- and nanofabrication. **a** Preparation of multilayer structures using LIFT technique^66^. Copyright 2012 John Wiley and Sons. **b** Chemical vapor deposition principle and prepared ferromagnetic graphene quartz fabric; b1Continuous roll-to-roll CVD growth system; b2 Preparation of ferromagnetic graphene quartz fabrics using chemical vapor deposition^74^. Copyright 2022 John Wiley and Sons. **c** Preparation of helical resonance structures using self-assembly technique; c1ALD growth method; c2 Schematic diagram of the preparation process^75^. Copyright 2020 Elsevier. **d** Uniform growth of two different carbon materials on graphene surface by chemical vapor deposition; d1 Schematic of a strained bilayer film rolled into a tube; d2 Schematic of the formation of a rolled carcass tube with a metal resonator; d3 Scanning electron microscope image of a parallel tubular chiral bi-directional anisotropic metamaterial^79^. Copyright 2017 Springer Nature

‘Bottom-up’ approaches start at the microscopic scale^57^, while the ‘top-down’ approach starts from the atomic or molecular level. Various entities are constructed by controlling the interaction forces between atoms, molecules^58,59^ and other nano-objects to form electromagnetic metamaterial structures^60–62^. ‘Bottom-up’ approaches allow for precise control of individual atoms or molecules, enabling the creation of highly precise, self-assembling, and structurally complex tiny metamaterial structures^63,64^. Figure 3 shows a common bottom-up process for processing flexible electromagnetic metamaterials.

The laser-induced forward transfer (LIFT) technique is a method used for patterning that employs photonic momentum to transfer material from one substrate (donor) to another (acceptor) after laser irradiation^31,65^. The process works by ejecting material from the film when the incident energy exceeds a coating-specific threshold, propelling it toward the receiving substrate. This technique allows for the formation of complex 2D and 3D patterns, with the writing speed typically determined by the laser’s repetition rate. The movement of the source and receiving substrates, or the scanning and modulation of the laser beam, facilitates pattern formation. High-resolution patterns are created from the individual printed voxels generated during the laser transfer process. In 2012, Tseng et al. proposed the LIFT technique based on the creation of the highly fluxed and efficiently fabricated cyclic multilayers of plasmonic metamaterials^66^.

Chemical Vapor Deposition (CVD) is a method used to deposit thin films or coatings on solid surfaces through gas-phase chemical reactions at high temperatures^67^. In the fabrication of electromagnetic metamaterials, CVD allows for precise control over the microstructure. By manipulating the arrangement and scale of the deposited material during the CVD process, the specific properties of the metamaterial can be tailored. Atomic Layer Deposition (ALD) shares a similar growth principle with traditional CVD, as it is also a vapor-phase growth technology^68,69^. However, ALD offers even greater precision in thin-film control, enabling the production of complex nanostructures with atomic-level thickness and uniformity^70,71^. Unlike CVD, ALD deposits precursors alternately, with the chemical reaction of each new atomic layer directly depending on the previous one. This method deposits only one atomic layer at a time^72,73^. The self-limiting nature of ALD allows for conformal film deposition without pinholes, enabling precise control of film thickness by adjusting the number of deposition cycles. In 2022, Xie et al. prepared a lightweight, flexible, and ferromagnetic material called graphene quartz fiber fabric (FGQF) using CVD^74^. In 2020, Xu et al. designed and synthesized two novel layered structures using an ALD-assisted method^75^.

Self-assembly technology involves the spontaneous organization of basic structural units—ranging from molecules to nanomaterials, and even to micrometer-scale or larger materials—into specific structures according to predetermined rules or external stimuli^24,76^. This process allows for precise modulation of electromagnetic waves at the microscopic scale^77,78^. In 2017, Prinz et al. proposed a new type of structure^79^. It consists of a GaAs/InGaAs strain thin film layer that is realized as an array of self-assembled elements on a GaAs substrate.

At present, various micro-nano manufacturing technologies are employed to fabricate flexible electromagnetic metamaterials. Table 1 summarizes and compares the currently common processing techniques for preparing flexible electromagnetic metamaterials. Advances in nanoimprinting technology are enhancing processing accuracy and efficiency, particularly for producing small and high-precision metamaterial structures. Similarly, improvements in 3D printing technology are expected to boost its capability for multi-material printing and high-resolution manufacturing. This progress will facilitate the production of more complex flexible electromagnetic metamaterials and help reduce production costs. Additionally, self-assembly technology will continue to evolve, enabling the creation of more intricate and sophisticated flexible electromagnetic metamaterials. Nonetheless, achieving large-area, high-precision, and cross-scale manufacturing remains a top priority for future development.

**Table 1:** Summary of the processing methods for the preparation of electromagnetic metamaterials

Processing method	Machining precision	Advantages	Disadvantages	Processing speed	Processing cost	Processing dimensions
Lithography^34^	High processing accuracy	No diffraction limit, processing technology is controllable	Large area processing is difficult	High processing efficiency	Low manufacturing cost, large-scale production is possible	Line width <100 nm
3D-printing Technology^46^	High resolution and precision	Flexible production of any topology	Limited to surface structure preparation	High printing efficiency	Low processing cost	Highest resolution down to 100 nm
Inkjet printing technology^51^	High processing accuracy	Controllable mode, low material consumption	Need to adapt to different deposition environments	High time efficiency but low output	No complicated processing, low cost	Minimum line width less than 150 μm
Femtosecond Laser Direct Writing Processing Technology^58^	High-precision microstructure manufacturing	Processing accuracy exceeds the diffraction limit, non-contact processing	Cannot process large areas, low processing efficiency	Low processing cost	Relatively slow processing speed	Processing accuracy less than 100 nm
Laser-induced Forward Transfer (LIFT)^68^	High-resolution	Rapid molding, easy operation	Processing materials are limited, processing cycle is long	Low processing cost	Relatively high processing rate	Processing size is within the range of 100–200nm
Chemical vapor deposition (CVD)^76^	High precision	Uniform processing, multi-level processing is possible	Limited to film structures, Usually requires high temperature environment	Faster processing speed	High processing cost and complicated operation	Thickness greater than 10 nm is difficult to control
Atomic Layer Deposition (ALD)^77^	High accuracy	Good flatness, Preparation of multi-layer complex structures	High processing cost and low deposition efficiency	Low deposition efficiency	Slow deposition rate	0.07– 0.1 nm
Self-assembly Technology^81^	High processing accuracy	Simple operation, relatively low material cost, fast assembly, and controllable layered structure	Only simple patterns can be prepared, and the process is difficult to monitor	Low preparation cost	High processing efficiency	Achieve processing size of 500 nm and below

Firstly, the current preparation processes for flexible electromagnetic metamaterials are limited to small areas. A major trend will be the development of methods for large-scale manufacturing. For instance, Park et al. utilized a one-step printing technique to achieve high-throughput production of large-sized metamaterials^80^. Swain et al. applied thermal tape transfer technology to fabricate large-scale (100 × 100 μm²) metamaterials with ultra-thin thickness (approximately 40 nm)^81^. However, further exploration is needed to adapt these methods for flexible electromagnetic metamaterials. Secondly, flexible metamaterials find applications in smart wearable devices, flexible electronics, biomedicine, and other fields. Practical applications must address issues such as manufacturing costs, output, and productivity. Jing et al. integrated metamaterials with computer vision technology, enabling flexible arrangement of optical devices in various scenarios and reducing production costs^82^. Kang et al. demonstrated the use of soft lithography to achieve large-area imaging on flexible substrates^83^. This method, due to its simple processes (molding, stamping, etc.) and use of low-cost organic/polymer materials, significantly reduces production costs.

In summary, further innovation and optimization of manufacturing technologies for flexible electromagnetic metamaterials are essential. The focus should be on achieving large-area, high-precision processes, improving processing technology, reducing production costs, and enhancing efficiency. To advance the field, exploring emerging manufacturing methods is crucial.

## The controllable ability of flexible electromagnetic metamaterials in different bands

Electromagnetic absorbing metamaterials are synthetic materials designed to effectively absorb electromagnetic waves and convert electromagnetic energy into other forms, such as heat ^84^. However, the surfaces of most objects that require electromagnetic shielding or protection from electromagnetic interference are often irregular, and electromagnetic waves can be incident from various directions. To address this, conformable electromagnetic metamaterial absorbers using flexible substrates have been developed. As the name suggests, flexible electromagnetic absorbing metamaterials possess toughness and can conform to the surfaces of irregular objects. Unlike traditional electromagnetic absorbing metamaterials, these flexible variants can be easily adhered to various complex surfaces, providing superior adaptability.

The flexibility in electromagnetic metamaterials is typically achieved through the dielectric isolation layer within their three-layer structure. In 2008, N. I. Landry pioneered the design and realization of a metamaterial absorber featuring a sandwich structure^85,86^. This absorber consists of three layers: an upper electric resonant ring that facilitates electric field coupling, a middle dielectric layer, and a lower metal wire. When exposed to an incident wave, the electric resonant ring and the lower metal wire generate antiparallel currents, providing magnetic field coupling. The electric and magnetic resonances within the metamaterial absorber can be tuned by adjusting the size parameters of each layer and the periodic arrangement of the resonant ring. The dielectric constant and magnetic permeability exhibit large imaginary components, ensuring impedance matching and a high absorption rate for electromagnetic waves. The middle layer, composed of a flexible, bendable, and conformal dielectric material, offers both toughness and wave absorption capabilities. This makes it particularly advantageous for covering irregular surfaces to absorb electromagnetic waves, and it also enhances the absorption of waves incident at large angles.

Since the structure of flexible electromagnetic metamaterials is artificially designed, their absorption performance can be adjusted, optimized, and modified by altering the materials and construction methods. This allows absorbers to achieve functional characteristics such as ultra-narrowband ^87^, multi-band ^88^, broadband ^89^, cross-band ^90^, flexibility ^91^, transparency ^92^, or tunability ^93^. Additionally, most flexible electromagnetic metamaterials are composed of periodic units with microstructures arranged in a plane. The scale of these periods and microstructures is related to the operating wavelength. By adjusting the period scale and designing the internal structure, various metamaterial absorbers can be developed to operate in different wavelength regions, including microwave ^94^, terahertz ^95^, and infrared ^96^.

The design methodology for flexible electromagnetic metamaterials was initially applied to the microwave band and later expanded to other bands, benefiting from the ease of cross-band research. Terahertz absorbers were first realized in 2008 ^97^, followed by near-infrared absorbers in 2009 ^98^. Since then, numerous designs and modulations of electromagnetic metamaterials across different bands have been proposed by researchers, emerging rapidly and abundantly.

The microwave band (0.3–300 GHz) is the most commonly used frequency range in modern wireless network systems, communications, radar, and other fields. It is characterized by penetration, heating selectivity, low thermal inertia, non-ionization, and information transmission. Flexible electromagnetic metamaterial absorbers, being sub-wavelength structures, typically operate in the millimeter scale within the microwave band, which makes them cost-effective to produce. This is currently one of the most important areas of research. The working wavelength can be controlled by adjusting the scale of the flexible electromagnetic metamaterial structure. When the scale of the metamaterial absorber reaches the micrometer or nanometer level, it can absorb terahertz and infrared waves through careful design and arrangement. The terahertz band, with wavelengths ranging from 30 µm to 3000 µm and a frequency range of 0.1 THz–10 THz, shares similarities with both microwave and optical theories, but also possesses unique properties. Terahertz waves exhibit both electronic and optical characteristics, leading to high penetration, particularly through non-metallic materials, and high resolution, which allows for microscopic imaging with excellent clarity. The infrared spectrum, typically covering the frequency range of 300 GHz–430 GHz, is characterized by high frequency and short wavelength. Metamaterial absorbers that function in the terahertz and infrared bands often have a period scale in the hundreds of nanometers. In the infrared band, metamaterials usually employ artificially designed microstructures made of metals or insulators to manipulate electromagnetic waves. With the advancement of multi-spectral composite detection technology, cross-band flexible electromagnetic metamaterials have begun to emerge. The design of these metamaterials focuses on absorbing high-frequency microwaves and sub-terahertz waves, or near-infrared, high-frequency terahertz, and visible light. However, there is currently some overlap in the operational frequency domains, and it remains challenging to achieve microwave-terahertz-infrared cross-frequency absorption with a single material or unit array. Most designs involve multi-layer or stacked structures, with narrow bandwidth or extremely low operational bandwidth.

### The microwave frequency band

**Fig. 4:**
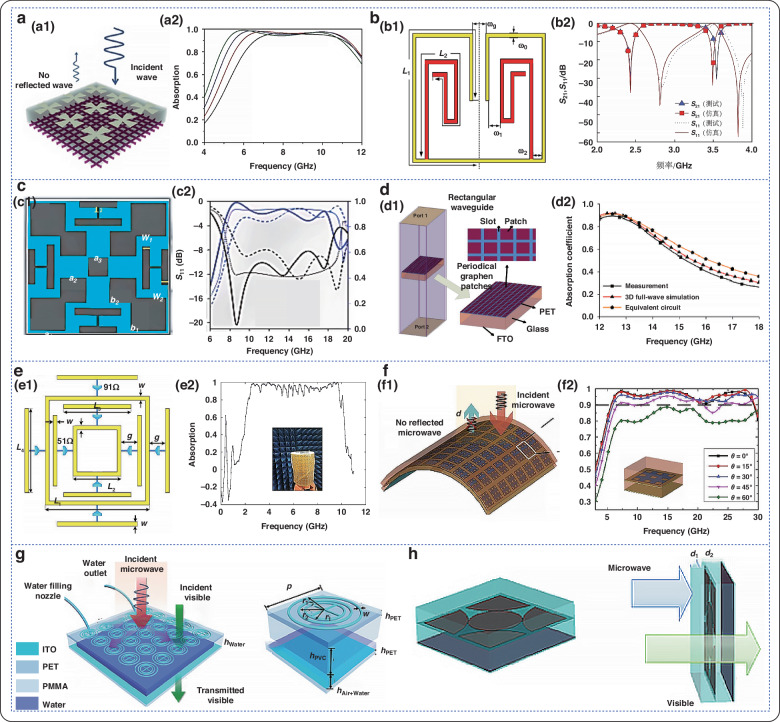
Modulation of flexible electromagnetic metamaterials in the microwave band. **a** Microwave absorber; a1 Schematic diagram of the microwave absorber; a2 Simulated absorption curves at different widths^106^. Copyright 2014 American Chemical Society. **b** Hairpin-type open resonance ring absorber; b1 Schematic diagram of the structure; b2 Microwave frequency response characteristics of structured metamaterials with the same incidence angle^107^. Copyright 2015 Acta Physica Sinica. **c** Prepared flexible graphene absorber; c1 Schematic structure; c2 Absorption curve of this absorber^108^. Copyright 2016 Springer Nature. **d** Configuration of the absorber and waveguide used for measurements; d1 Schematic of the structure; d2 Absorption profile of the absorber^109^. Copyright 2017 IEEE. **e** Schematic of the simulated absorber; e1 Cell structure parameters of the designed absorber; e2 Absorption measurements attached to a cylinder^110^. Copyright 2020 MDPI. **f** Geometry of PET coated with patterned ITO film; f1 Simulation of MMA cell at normal incidence angle; f2 Simulated absorbance of MMA cell at different polarization angle^111^. Copyright 2021 IOP Publishing. **g** Schematic of a water-based tunable transparent metamaterial absorber^112^. Copyright 2021 RSC Pub. **h** Periodic square ITO patch structure cell with high fill ratio characteristics^113^. Copyright 2023 SPIE

The microwave frequency band encompasses electromagnetic waves ranging from 300 MHz to 300 GHz. In the design and manufacturing of microwave devices, absorbers typically employ subwavelength-scale structural designs. This allows for efficient absorption of electromagnetic waves within a smaller physical size. Microwave absorbers are known for their high absorptivity, effectively converting incident electromagnetic wave energy into heat or other forms of energy^99^. Given that the size of microwave band absorbers is relatively large, usually at the millimeter scale, the manufacturing processes involved are well-developed and cost-effective. Flexible electromagnetic metamaterials are versatile and can be utilized in designing common microwave devices such as frequency selective surfaces ^100^, absorbers, highly directional antennas^101^, couplers ^102^, power dividers ^103^, bandpass filters ^104^, broadband phase shifters, and flat focusing lenses ^105^. These applications contribute significantly to enhancing the performance of microwave devices. Figure 4 shows the modulation of flexible electromagnetic metamaterials in the microwave frequency band.

In 2014, Jang et al. designed an optically transparent, flexible, and polarization-dependent broadband microwave absorber^106^. A simulation was used to calculate the absorption rate of the absorber, which was found to be greater than 90% in the frequency range of 5.8–12.2 GHz. In 2015, Liu et al. designed a miniaturized dual-band metamaterial based on a hybrid arrangement of unit cells with open resonance rings^107^. The insertion loss was -15 dB at 2.4 GHz with a relative bandwidth of 4.2% (2.37–2.47 GHz) and -29 dB at 3.5 GHz with a relative bandwidth of 2.3% (3.45–3.53 GHz). In 2016, Huang et al. designed a nanomaterial for a broadband radar absorber^108^. Nanomaterials are based on a modified second-order saltire cross structure and four H-shaped coupling additives. It features printed graphene nanosheet conductive patterns for impedance matching and metal grounding. Modeled and simulated using CST, it provides effective absorption in the range of 10.4 GHz–19.7 GHz with a fractional bandwidth of 61.8% at a thickness of approximately 2 mm. In 2017, Da et al. designed a transparent microwave absorber using patterned graphene^109^. Researchers transferred graphene to a polyethylene terephthalate (PET) film to create a transparent absorber that operates in the Ku band. Overall, the transmittance of the graphene/PET film is approximately 80% over the whole optical range. At the Ku-band, the absorption coefficient reaches 90%, and at 12.6 GHz, the absorption coefficient also reaches 90%. In 2020, Fan et al. proposed an ultrawideband flexible absorber^110^. A sandwich design absorber was simulated using CST. From the simulation results, it is clear that the absorber has a bandwidth of 2.55-10.07 GHz with an absorption rate of more than 90% and a relative bandwidth of more than 119.2%. In 2021, Zhao et al. proposed an ultrabroadband and wide-angle optically transparent flexible metamaterial absorber^111^. It consists of a multilayer structure with a transparent polyvinyl chloride (PVC) layer and a flexible metamaterial absorber attached to it. In addition, the multilayer structure includes periodic ITO patch arrays attached to a polyethylene terephthalate film. The absorption of the incident wave remains above 90%. In 2021, Zhang et al. proposed a tunable transparent metamaterial absorber with high optical transparency and broadband microwave absorption performance^112^. With an absorption rate of more than 90%, the absorber can achieve ultrabroadband absorption in the range of 5.8–16.2 GHz. In 2023, Xu et al. proposed an optically transparent electromagnetic metamaterials structure with broadband microwave absorption^113^. It is composed of different layers on transparent conductive ITO deposited on transparent PET substrates with different surface resistances. CST software was used to simulate the structure, which was found to have a high absorption rate in the 8.7–39.2 GHz microwave band, with absorption greater than 90%.

### The terahertz band

**Fig. 5:**
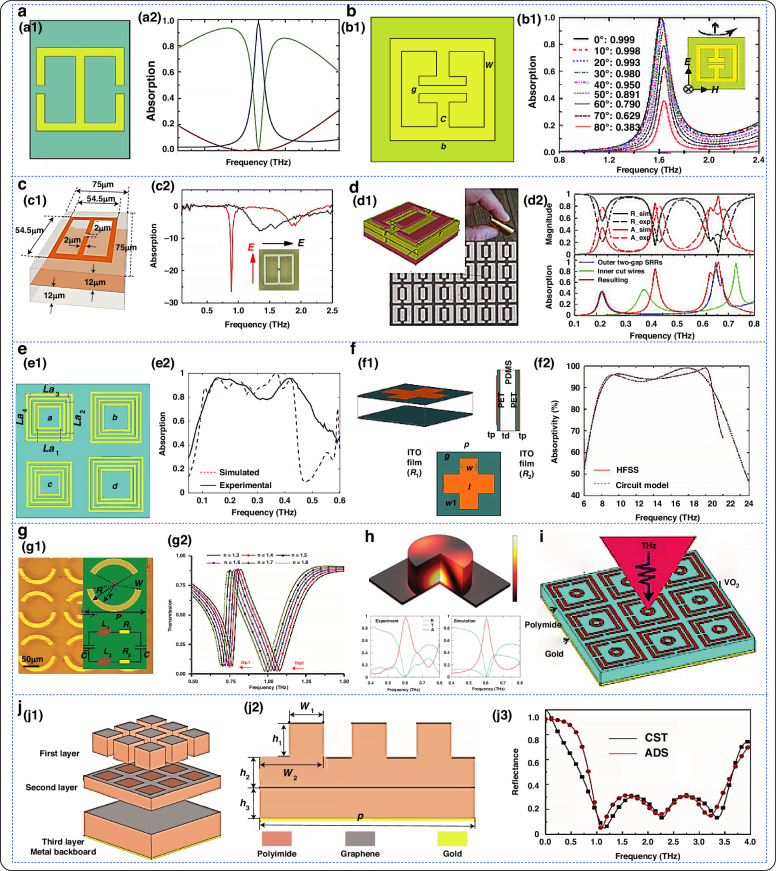
Modulation of flexible electromagnetic metamaterials in the terahertz band. **a** Terahertz absorber; a1 Schematic diagram of the terahertz absorber; a2 Absorption rate of an electrically resonant ring in the 1.12 terahertz band^122^. Copyright 2008 OPTICA. **b** Terahertz metamaterial absorber; b1 Schematic of the absorber; b2 Simulation of the frequency function for different angles of incidence^123^. Copyright 2008 The American Physical Society. **c** Metamaterial absorber; c1 Cell layout and dimensions; c2 Measured reflection coefficients at normal sample incidence^124^. Copyright 2012 OPTICA. **d** Simulation of a metamaterial absorber^125^. Copyright 2013 OPTICA. **e** Dual-frequency absorber; e1 Schematic structure; e2 Comparison of absorption spectra^128^. Copyright 2018 AIP Publishing. **f** Terahertz absorber; f1 Schematic of thin-film sensor; f2 Spectrogram of terahertz bands^129^. Copyright 2020 John Wiley and Sons. **g** Three-dimensional view of the absorption spectrum of a single cell; g1 Structural schematic; g2 Relationship between transmittance and refractive index of analytes^130^. Copyright 2020 Elsevier. **h** Geometry of the absorption cell^131^. Copyright 2021 OPTICA. **i** Perspective array of the proposed structure^132^. Copyright 2022 Frontiers in Physics. **j** Simulated terahertz absorber; j1 Schematic of the three-dimensional structure; j2 Planar geometry; j3 Absorption profile in terahertz bands^133^. Copyright 2022 OPTICA

Terahertz waves refer to the frequency band of the electromagnetic spectrum between microwaves and the infrared region. The frequency ranges from 0.1 THz to 10 THz, and the wavelength ranges from 3 to 0.03 mm^114^. Among other advantages, coherence^115^, high resolution^116^, broadband^117^, and low energy^118,119^ are available. Additionally, terahertz waves have high penetrability, high imaging resolution, wide bandwidth, and high-speed communication capabilities. In recent years, terahertz research and applications have rapidly developed and become widely used in terahertz imaging, nondestructive testing, and biosensing^120,121^. Figure 5 shows the modulation of flexible electromagnetic metamaterials in the terahertz frequency band.

In 2008, Tao et al. proposed a metamaterial absorber for terahertz frequencies^122^. After continuous optimization and improvement, a 70% absorption rate was achieved at 1.3 THz. An absorption rate of 0.97 at 1.6 THz was experimentally demonstrated. In the same year, A. C. Strikwerda et al. designed and realized the first terahertz metamaterial absorber with a metal plate as the bottom layer and a flexible polyimide (PI) layer as the intermediate dielectric. In 2011, Iwaszczuk et al. designed a flexible metamaterial thin film for stealth applications at terahertz frequencies MMA^123^. An instrument for determining the bistable radar cross section (RCS) at broadband terahertz frequencies was also developed. The results demonstrated a nearly 400-fold reduction in the radar cross-section at the design frequency of 0.87 THz. In 2013, Riadyahiaoui et al. designed an open-ended split ring resonator (SRR) structure for use in terahertz band metamaterials^124^. The intermediate dielectric layer is a flexible PI substrate. The maximum absorption rate in this study is approximately 80% at submillimeter wavelengths in the 0.1–1 THz band. In 2015, Shan et al. proposed a metamaterial-based ultrathin flexible dual-band absorber at terahertz frequencies^125^. The intermediate dielectric layer of the absorber is made of a flexible material with a thickness of 25 μm. According to the study, the absorber is flexible with an intermediate dielectric layer of PI. Using PI structure, two resonant absorption peaks were observed at 0.41 THz and 0.75 THz, with absorption rates of 92.2% and 97.4%, respectively. In 2017, Liu et al. demonstrated a nanoparticle-doped thin-film sensing method based on terahertz metamaterials^126^. Polyvinyl alcohol (PVA) films doped with various concentrations of magnetic nanoparticles were rotated and coated onto the surface of terahertz metamaterials. The concentration sensing sensitivity reached 3.12 GHz/0.1%. In 2018, Shen et al. presented a flexible and broadband terahertz metamaterial absorber that consists of metal and dielectric materials, specifically gold and polyethylene glycol ester (PEN), respectively^127^. Experimentally observed broadband properties in the terahertz band showed an average absorption of 88% for TE polarization in the range of 0.63 to 1.34 THz. In 2020, Tayde et al. designed a transparent broadband absorber with a flexible structure using polydimethylsiloxane as a dielectric substrate^128^. The proposed absorber exhibited an absorption greater than 90% in the frequency range from 8.00 to 20.70 GHz, covering the entire X and Ku bands under normal incidence. In 2020, Cheng et al. proposed a planar array Fano asymmetric open-ring resonator fabricated on a flexible PI substrate^129^. The resonators exhibited refractive index sensitivities of 160 GHz/RIU and 240 GHz/RIU near the 0.81 and 1.13 THz transmission tilts, respectively. In 2021, Wang et al. improved the structure of the absorber unit, which is composed of the following components from top to bottom: gold, a dielectric (PI), and a metal absorber^130,131^.According to the experimental results, the all-dielectric hypersurface exhibited an absorbance of up to 96% at 603 GHz. In 2022, Abdulkarim et al. proposed a novel broadband metamaterial absorber based on a vanadium dioxide (VO2) resonator coated on a flexible polyamide substrate^132^. The proposed metamaterial structure has two distinct peaks at 0.88 and 1.42 THz. In 2022, Huang et al. designed a laminated broadband absorber using a composite structure of graphene and PI^133^. The simulation results indicate that the absorber has an absorptivity of more than 90% in the 0.86–3.54 THz region.

### The infrared band

**Fig. 6:**
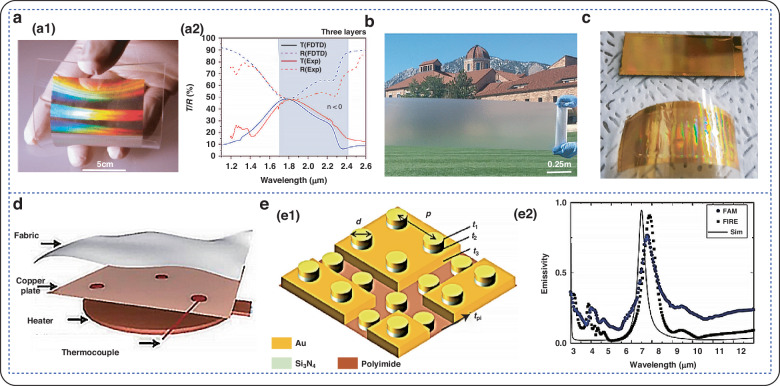
Modulation of flexible electromagnetic metamaterials in the visible band. **a** Broadband absorber; a1 Schematic of the absorber at visible frequencies; a2 Simulated absorption spectra^136^. Copyright 2011 Springer Nature. **b** Schematic of the absorber based on patterned graphene film^137^. Copyright 2017 Science. **c** Visible absorber; c1 Schematic of MPA with alternating ITO and photoresist layers; c2 Transmission of metamaterials at visible wavelengths^138^. Copyright 2021 American Chemical Society. **d** Absorber consisting of a five-layer MIM stacked array; d1 Schematic of the metamaterial absorber; d2 Simulated absorption and reflection spectra^139^. Copyright 2021 Science (e) MMA absorber composed of ITO, PMMA, and PET; e1 Schematic of the structure; e2 Reflection loss curves^140^. Copyright 2022 John Wiley and Sons

Infrared light is the frequency range between visible light and microwaves in the electromagnetic spectrum, with a frequency range of 300 GHz–430 THz^134,135^. Advancements in infrared thermal imaging and detection technology have considerably improved the damage performance and battlefield survivability of weapons and equipment, highlighting the need for continued improvement. Figure 6 shows the modulation of the flexible electromagnetic metamaterial in the infrared band.

In 2011, Chanda et al. used nanoimprinting techniques to transfer inked materials onto rigid or flexible substrates^136^. Experimental measurements and simulations have demonstrated that large-area 3D metamaterials fabricated in this manner exhibit a strong negative refractive index in the near-infrared spectral range. Additionally, nanoscale 3D NIMs (>75 cm^2^) fabricated in this manner also exhibit a strong negative refractive index in the near-infrared spectral range. In 2017, Zhai et al. introduced a new glass-polymer hybrid material^137^. It appears to contain resonant polar dielectric microspheres randomly embedded in a polymer matrix. As a result, the material is transparent in the visible region and has an infrared emissivity greater than 0.93 over an atmospheric window. In 2021, Namkyu-Lee and colleagues introduced a flexible thermal infrared camouflage material that allows for selective energy dissipation in a supersonic flow field. To enhance the flexibility of the material, researchers have replaced the dielectric layer with a PI polymer^138^. Researchers quantified the infrared cloaking performance of the FTCM and found that the emissivities in the detection band were 0.12 (3-5 μm) and 0.16 (8-14 μm). In 2021, Ceng et al. developed a multilayer composite^139^. Hyperfabric consists of a titanium oxide composite with a laminated polytetrafluoroethylene multilayer. The massively woven hyperfabric could provide high emissivity (94.5%) in the atmospheric window (3-5 μm). In 2022, Lee et al. developed multispectral camouflage by assembling flexible infrared emitters (FIREs) and flexible microwave absorbers (FMWAs) using an intermediate layer^140^. FMWAs are passed through an intermediate layer to enhance the high absorption bandwidth (>0.9) in the S (2–4 GHz), C (4–8 GHz) and X (8–12 GHz) bands.

### Multiband compatibility

**Fig. 7:**
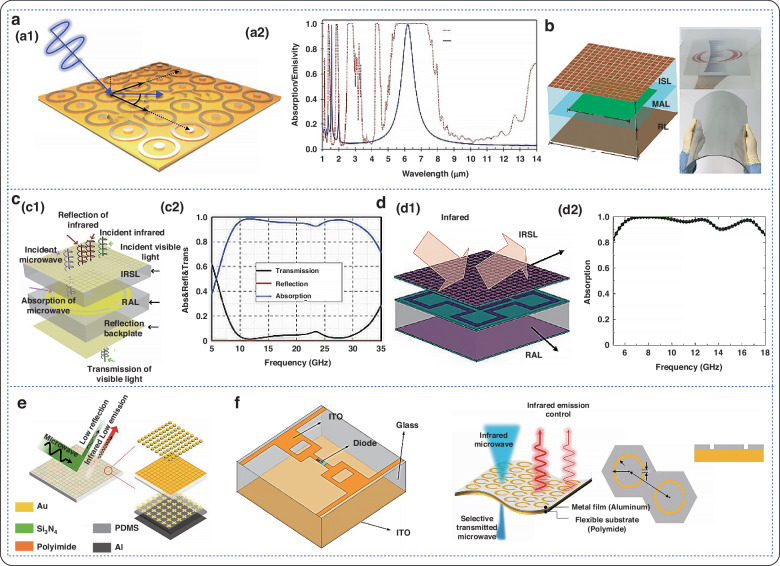
Multi-band modulation of flexible electromagnetic metamaterials. **a** Dual-band perfect absorber; a1 Structural diagram; a2 Spectral characteristics of dual-band perfect absorber^141^. Copyright 2017 Springer Nature. **b** Simulated microwave infrared unit cell bistable structure^142^. Copyright 2019 John Wiley and Sons. **c** Schematic diagram of realizing microwave absorption, low infrared emission and optical transparency; c1 Structural diagram; c2 Absorption, reflection and transmission diagram at normal incidence^143^. Copyright 2021 OPTICA. **d** Side view of radar-infrared dual stealth structure; d1 Structural diagram; d2 Absorption rate at different azimuth angles^144^. Copyright 2021 IOP Publishing. **e** Flexible assembly consisting of a flexible infrared emitter and a microwave absorber^145^. Copyright 2022 AIP Publishing. **f** Schematic diagram of a single cell consisting of a three-layer structure^146^. Copyright 2023 John Wiley and Sons

The difficulty in achieving multi-band compatible camouflage lies in the fact that the emissivity spectra of different camouflage technologies are completely different and even inherently competitive. The emergence of metamaterials provides new possibilities for achieving multi-band emissivity modulation by adjusting electromagnetic behavior through microstructures. Figure 7 shows that the flexible electromagnetic metamaterial is compatible with modulation in multiple frequency bands.

**Fig. 8:**
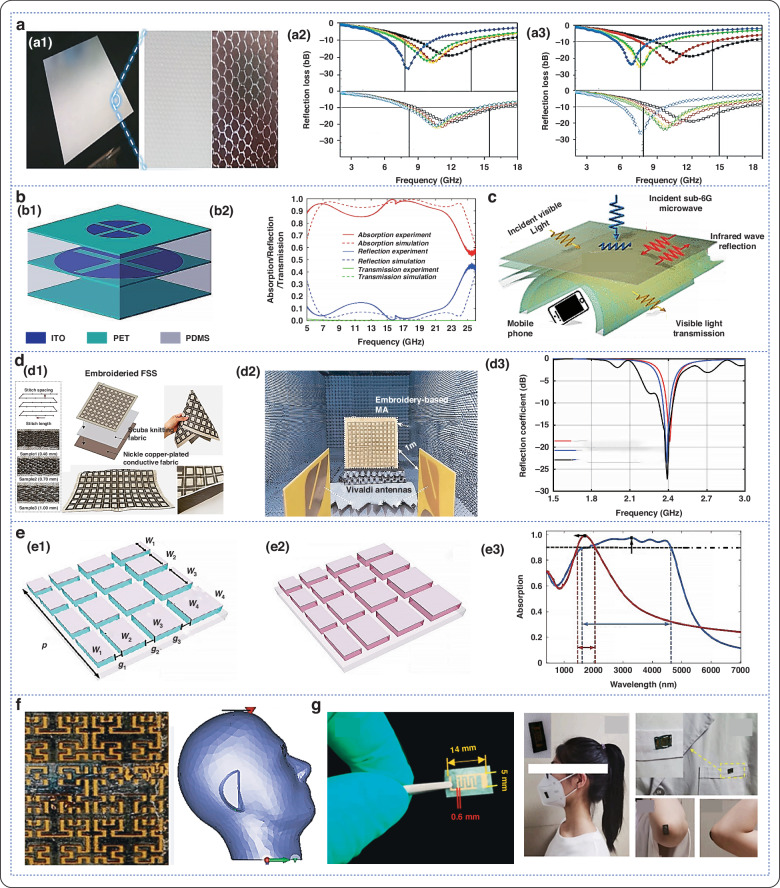
Application of the flexible electromagnetic absorption metamaterials. **a** Double-layer composite coating based on moth-eye-like hexagonal anti-reflection structure; a1 Sample image; a2 Reflection loss curve of 10μm double-layer composite coating in the 2–18 GHz frequency band; a3 Reflection loss curve of 20 μm double-layer composite coating in the 2–18 GHz frequency band^152^. Copyright 2022 John Wiley and Sons. **b** Microwave broadband transparent metamaterial anti-reflection absorber (OTMA) based on ITO; b1 Geometric structure parameters; b2 Experimental and simulated curves of absorption, reflection and projection^153^. **c** Working principle of electromagnetic shielding of metamaterial absorber^159^. Copyright 2022 American Chemical Society. **d** Flexible electromagnetic shielding material absorber based on embroidery; d1 Sample image with different embroidery densities; d2 Sample measured in an anechoic chamber; d3 Comparison between simulated and measured reflection coefficients^14^. Copyright 2022 Elsevier. **e** Schematic diagram of metamaterial ultra-wideband absorber; e1 Insulating phase; e2 Metal phase; e3 Calculated absorbance spectrum^163^. Copyright 2019 OPTICA. **f** Wearable antenna sensor^9^. Copyright 2021 MDPI (**g**) Schematic diagram of flexible PAN/MWCNT/PANI sensor^167^. Copyright 2024 American Chemical Society

In 2017, Kim et al. proposed a perfect absorber based on a triple-layer structure of Ag-PI-Ag metamaterials to achieve dual-band stealth compatible with mid-wave infrared (MWIR) and longwave infrared (LWIR) radiation, as well as a 1.54 μm infrared laser (IRL)^141^. Based on this study, the proposed absorber reduced the 1.54 μm IR-LWIR scatter by more than 90% and suppressed the MWIR and LWIR signals by more than 92%. In 2019, Luo’s team designed a metamaterial structure that is transparent and flexible and has dual-stealth capabilities for microwave and infrared frequencies^142^. It consists of three layers of ITO film and two layers of PVC substrate. The ISL layer, composed of ITO square patch arrays, achieves high microwave transmittance and low infrared emissivity. The experimental results demonstrate that the metamaterial structure absorbs 90% of the broad bandwidth of 7.7–18 GHz, has low infrared emissivity in the atmospheric window range, and is optically transparent at approximately 30%. In 2020, Gao et al. designed and fabricated optically transparent metamaterials with infrared-radar compatible stealth properties^143^. These materials are based on ongoing, in-depth research into single-band stealth technology. Transparent PET and ITO materials were utilized to create a flexible metamaterial consisting of an infrared shielding layer (IRSL) and a radar absorbing layer (RAL). In the infrared atmospheric window, the results indicate an absorption rate of over 90% and an emissivity of 0.3% in the 8.7-32 GHz range. In 2022, Xu et al. from the same group utilized the optically transparent materials ITO and PET to design a radar-infrared-visible-compatible stealthy hypersurface with an ultrawideband^144^. The microstructure consisted of an upper IRSL and a lower RAL, similar to their previous work. The emissivity of the metamaterials was approximately 0.46 in the infrared band from 3 to 14 μm. Lee et al. designed flexibly assembled metamaterials that are infrared-microwave stealth-compatible^140^. Investigators achieved this by assembling FIREs and FMWAs using PI as a substrate for multispectral cloaking. An intermediate layer in the FMWA provides broadband enhancement with high absorption ( > 0.9) in the S (2–4 GHz), C (4–8 GHz), and X (8–12 GHz) bands. In 2022, Yang et al. prepared a transparent, electrically tunable, wave-absorbing material for electromagnetic protection and stealth^145^. In visible light, the transmittance of the sample was 80.23%. It exhibits high-performance electromagnetic shielding properties with an effectiveness of more than 30 dB in the 2.6–3.95 GHz range. Consequently, the metamaterial enables transparent radar-infrared stealth compatibility. In 2023, Nam et al. developed a flexible camouflage material that is compatible with longwave-microwave multiband frequencies^146^. The optical infrared properties of polydimethylsiloxane (PDMS), specifically its tunable emission, can be effectively controlled while maintaining its microwave transmission characteristics. Finally, the metamaterial is composed of a flexible polydimethylsiloxane substrate, a PI film, and a patterned high-conductivity aluminum film. Overall, the experimental results show that the metamaterial has high emissivity in the long-wavelength infrared region, with an infrared tunable emission rate of 77.7% and a microwave transmittance of 93.8%.

## Applications of flexible electromagnetic absorbing metamaterials

### Electromagnetic metamaterials exhibit antireflection properties

Reflection and refraction typically occur when an electromagnetic or light wave encounters an interface consisting of two media with different optical properties^147,148^. Reflection is a common phenomenon in daily life. In nature, many objects cannot emit light on their own. What allows us to see these objects is that the human eye can perceive the visible light reflected from them. Currently, there are two main methods for stealth mechanisms based on metamaterials^1^: A reflective ultrathin stealth suit can be used, which manipulates the reflected wavefront by covering a layer of metamaterials on a folded reflective surface to simulate the reflected wavefront of a smooth mirror; however, specular reflection is achieved with this method^2^. An absorption-radiation-type ultrathin stealth suit can be used. It involves absorbing the incident wave with a lossy metamaterials, followed by radiating and adjusting the wavefront with a gainful metamaterials. Tuning the gain is the main challenge of this approach. Furthermore, a composite ultrathin cloak is utilized to achieve the stealth effect. In this case, the stealth effect can be achieved by using a transmissive metamaterials to couple the incident electromagnetic wave into a zero-refractive index material^149^. The electromagnetic wave then passes through the tunnel to the outgoing end and is controlled by the transmissive metamaterials. One of the main challenges of this method is the realization of zero refractive index materials^150^. For low-loss materials such as dielectrics, a new stealth method involves the use of metamaterials to allow the incident wave to pass directly through the dielectric surface without reflection or refraction. By allowing the wave to pass directly through the entire object, the dielectric effect can be achieved^151^. Figure 8 shows the application of flexible electromagnetic absorption metamaterials.

In 2022, Wang et al. successfully fabricated a double-layer composite coating based on a moth-eye hexagonal anti-reflection structure, utilizing flexible polyurethane (PU) as the substrate^152^. This double-layer composite coating achieved a maximum effective absorption bandwidth of 7.4 GHz in the S-Ku band and exhibited an infrared emissivity of 0.292. Simulation results indicate that the hexagonal periodic arrangement of the IRSL can disrupt the continuity of the electric field and induce a networked concentration of the electric field and surface current within the absorbing layer, thereby enhancing the wave absorption performance of the double-layer coating. In 2024, Wang et al. proposed a multilayer optically transparent metamaterial absorber (OTMA) based on ITO and PDMS^153^. By calculating the equivalent electromagnetic parameters, they analyzed the electromagnetic loss of the OTMA and found that it is attributed to the combined effects of electoral resonance and magnetic resonance. Within the frequency range of 6 GHz to 26 GHz, the structure achieves impedance matching, thereby minimizing reflection.

### Enhanced electromagnetic shielding

Electromagnetic shielding refers to the use of material properties to reflect and absorb electromagnetic waves, thereby eliminating or attenuating them^154,155^. Shielding effectiveness (SE) measures the performance of electromagnetic shielding materials and reflects the degree of effective shielding of electromagnetic waves. Due to the reduced intensity of reflected waves, electromagnetic waves can be reflected by shielding materials, thereby reducing their propagation^156^. Additionally, shielding materials can absorb the energy of electromagnetic waves, achieving a shielding effect. Materials contain specific components or structures that absorb electromagnetic waves and convert their energy into other forms. During the absorption process, electromagnetic waves are attenuated, which improves the material’s shielding effectiveness. A material’s shielding effectiveness rating index, or SE, reflects its ability to block electromagnetic waves. Typically, materials with higher shielding effectiveness values provide better protection against electromagnetic waves. Promising trends in the development of electromagnetic interference (EMI) shielding materials currently include multifunctionality, eco-friendliness, flexibility, and low reflectivity^157,158^. However, integrating multiple functions into a single material remains a challenge.

Li et al. presented a multiperformance electromagnetic shielding material for the 5 G millimeter-wave band^159^. The multilayer structure consists of a low-infrared emitting layer, a PVC compensating layer, and a microwave absorbing layer. An optically transparent medium is used as the substrate, and an optically transparent ITO thin film is used instead of a metal resonant structural unit to achieve optically transparent absorptive electromagnetic shielding. In 2022, Yang et al. introduced a flexible electromagnetic shielding material absorber based on embroidery^14^. Experimental results demonstrated that, with an appropriate embroidery density, the material achieved an optimal absorption rate of 99% at a frequency of 2.39 GHz. This material exhibits excellent electromagnetic shielding performance and represents a new type of shielding material that is flexible, lightweight, and offers high design flexibility.

### Temperature regulation—thermal imaging

In terms of temperature control, metamaterials can regulate the propagation and absorption of thermal radiation by modifying their surface morphology, thereby achieving temperature control^160^. Temperature control of a metamaterials involves changes in its surface structure at the microscale^161^. Altering the microstructure of the metamaterials changes the reflection, transmission, and absorption properties of thermal radiation. Precise control of this microstructure can be used to achieve the desired control of thermal radiation at a specific wavelength.

In 2009, Driscoll et al. created a gold open resonance ring array on a VO_2_ thin film^162^. The terahertz resonance wavelength could be modulated by controlling the temperature. This was accomplished by tuning the infrared spectral resonance to the local Joule effect of the overcurrent to stimulate its phase transition. In 2019, Lei et al. introduced a scalable and tunable broadband metamaterial absorber that utilizes a VO_2_ thin film with thermotropic phase transition properties^163^. Below the critical temperature of the phase transition, VO_2_ behaves as a low-loss dielectric material. However, when the temperature reaches and exceeds the critical temperature, VO_2_ gradually transforms into a high-loss metallic material. Temperature regulation enables dynamic switching between broadband absorption (1627–4696 nm) and narrowband absorption (1443–2066 nm) in the infrared band.

### Flexible electromagnetic metamaterials for wearable sensors

As demand for health monitoring, sports tracking, and convenience continues to rise, flexible wearable electronic sensors have become crucial components. Flexible electromagnetic metamaterials play a significant role in this field due to their softness, lightness, breathability, and stretchability. These materials offer unique advantages in biomedicine, gas sensing, and pressure sensing, aligning well with practical needs. In wearable biomedical applications, several key specifications must be met for antenna design, including small size, light weight, low power consumption, and flexibility^164^. Additionally, wearable antennas experience a significant drop in performance when positioned close to the human body, posing challenges for wireless communication^165^. Furthermore, electromagnetic waves absorbed by the human body can have adverse environmental and biological effects^166^. To mitigate these issues, antennas can be mounted on flexible or semi-flexible substrates. This approach helps maintain their resistance to physical bending and distortion, reducing the environmental impact.

In 2021, Ammar et al. designed a microstrip antenna for wearable biomedical applications^9^. The antenna was fabricated by printing silver nanoparticle ink onto a polymer substrate. To suit various miniaturized wireless biomedical devices, the antenna was reduced to a size of 20 × 10 mm². Experimental tests conducted on the human body demonstrated that the antenna’s gain was 1 dBi at 403 MHz, 1.24 dBi at 433 MHz, 1.48 dBi at 611 MHz, 2.05 dBi at 912 MHz, and 4.11 dBi at 2.45 GHz. These results indicate that the antenna has a low specific absorption rate (SAR) effect on the human body. In 2024, Dong et al. developed an ultra-flexible wearable gas sensor using an electrospun polyacrylonitrile (PAN) nanofiber network as the substrate^167^. The unique nanofiber network and strong bonding between the substrate and the sensitive material provide the sensor with exceptional toughness, flexibility, and gas permeability. Experimental results showed that the sensor exhibits high sensitivity to NH_3_ at room temperature, with a theoretical detection limit as low as 300 ppb. This research offers a new approach for creating reliable and high-performance wearable gas sensors.

## Conclusion

Flexible electromagnetic metamaterials in different frequency bands enhance the electromagnetic properties of conventional materials, creating new opportunities for microwave, terahertz, infrared, and multiband compatible technologies. This study has significant implications for electromagnetic stealth, electromagnetic shielding, and temperature regulation. In this overview, the modulation mechanisms of flexible electromagnetic metamaterials in different frequency bands are explained. Well-designed metamaterial structures in the microwave band can achieve negative microwave refraction, focusing, and stealth, with applications in radar, communications, and antennas. Metamaterials also have applications in the infrared band, such as thermal radiation modulation, infrared sensing, and infrared optics devices. These applications provide new ideas and methods for the development of infrared technology. Electromagnetic metamaterials, which inject new energy into the development of terahertz technology, are also widely used in imaging, sensing, communication, and other fields within the terahertz band. In various frequency bands, electromagnetic metamaterials have a wide range of applications. Optimizing the material structure and design methods can expand their application fields, promote related technologies, and drive innovation in electromagnetic shielding, temperature regulation, reflection reduction, information technology, optical engineering, and wireless communications. Nevertheless, it is essential that future research address challenges such as material preparation technology, performance stability, and the difficulty of large-scale production.
